# Recent Advances in Physicochemical Control and Potential Green Ecologic Strategies Related to the Management of Mold in Stored Grains

**DOI:** 10.3390/foods14060961

**Published:** 2025-03-12

**Authors:** Tianyu Sha, Yujie Lu, Peihuan He, Md Mehedi Hassan, Yehan Tong

**Affiliations:** 1 School of Grain Science and Technology, Jiangsu University of Science and Technology, Zhenjiang 212003, China; 2 Jiangsu Provincial Engineering Research Center of Grain Bioprocessing, Jiangsu University of Science and Technology, Zhenjiang 212003, China; 3 Anhui Province Key Laboratory of Functional Agriculture and Functional Food, Anhui Science and Technology University, Chuzhou 239000, China; 4 School of Materials Science and Engineering, Jiangsu University of Science and Technology, Zhenjiang 212100, China; 5 College of Ocean Food and Biological Engineering, Jimei University, Xiamen 361021, China

**Keywords:** grain storage, mold prevention, innovative mold inhibitors, physical prevention, chemical prevention, micro–nano prevention

## Abstract

Grain serves as an essential cornerstone for sustaining life and social stability. However, during storage grain is often invaded by mold, which leads to mildew issues. This problem diminishes nutrient content and food quality and raises safety concerns, including toxin production, which can cause serious economic losses and catastrophic market stability and national food security conditions. Accordingly, implementing effective measures to prevent and control mold is crucial for ensuring grain storage safety. This paper analyzes the molds that affect grain during storage, discussing their varieties, environmental needs, and potential hazards. It also expounds on corresponding prevention and control measures, including physical methods, chemical approaches, innovative mold inhibitors derived from microbes and plants, and micro–nano prevention and control technology. These measures demonstrate significant mold suppression by destroying the cell structure of mold or inhibiting its physiological processes. In particular, micro–nano technology enables the effective embedding and controlled release of active ingredients. It can prolong the release duration and enhance antibacterial stability, thus achieving more effective control effects. Furthermore, it can be concluded that these strategies provide a theoretical foundation to enhance the safety and efficiency of grain storage. Additionally, they assist in more effectively addressing mold-related challenges while ensuring food security.

## 1. Introduction

Grain constitutes an essential resource for human existence and progress. It acts as vital insurance for sustaining life and ensuring social stability. There are grain storage industries in many areas and countries around the world. However, during storage, external humidity, hot air, and cold airflow in granaries and temperature differences in the grain pile can cause water to transfer to the grain stored, which leads to a partial increase in moisture content [1]. Such environmental changes can trigger the development of mold populations and accelerate their growth, potentially resulting in significant quality losses and even safety issues, such as mycotoxin production [2]. This can damage various aspects of grain quality, including seed discoloration, flavor alterations, reduced germination rates, and an increase in fatty acid value. Fatty acid value is an important indicator of grain storage quality, and its increase signifies a decline in grain quality. In addition, the spoilage of grain can lead to a reduction in its safety for consumption and a deterioration in its processing characteristics [3,4]. According to official data from researchers investigating mildew after grain harvesting, mycotoxins are secondary metabolites of fungi that primarily contaminate grains such as corn, wheat, and barley. Statistics show that approximately 2% of crops lose their nutritional and economic value due to severe contamination, resulting in economic losses amounting to hundreds of billions of dollars. Additionally, the toxins produced by molds can be a risk to human and animal health, possibly even becoming life-threatening [5]. Therefore, preventing and controlling molds in grain storage is the most important aspect. This can be effectively achieved through physical or chemical prevention and control technologies, innovative grain storage mold inhibitors, and micro–nano prevention and control technology. As society evolves, people’s awareness of environmental protection has gradually increased. Thus, the methods for preventing and controlling molds must be further optimized toward greater efficiency and sustainability.

This paper summarizes and elaborates the latest research on mold prevention and control. It focuses on common molds and their hazards during grain storage, prevention and control techniques, and innovative mold inhibitors for grain storage, aiming to provide more ideas for reducing the probability of mold occurrence and improving grain storage quality.

## 2. Common Molds and Their Hazards During Grain Storage

### 2.1. Types of Molds That Contaminate Cereal Crops

Molds exhibit the typical characteristics of fungi, being threadlike and reproducing with spores. Reports indicate that about 200 species of molds have been isolated from grains, among which 26 species belong to the genus *Aspergillus*, 67 species belong to the genus *Penicillium*, 30 species belong to the genus *Mucorales*, and 15 generally belong to the genus *Chaetomium* and *Filamentous Spores*. The common molds detrimental to grains primarily include *Aspergillus*, *Penicillium*, and *Fusarium* [6]. Additionally, based on growth characteristics, grain storage molds can be classified into field-type and storage-type molds, with a competitive succession occurring between the two. Generally, the mold community shifts from field-type to storage-type as storage time increases. Field fungi, like *Fusarium*, mainly infest freshly harvested grain, but after 2–4 months of storage, these field fungi gradually disappear. Several months later, *Aspergillus* and *Penicillium* spp. replace field fungi as the main fungal groups [7,8].

During prolonged grain storage, the microorganisms associated with grains change in both type and quantity over time. Initially, *Fusarium graminearum* and *Aspergillus* dominate in the grain kernels. However, as storage duration increases, the composition and proportion of molds within the grain pile evolve. After a period of time, the prevalence of *Penicillium*, *Cladosporium*, and *Aspergillus niger* diminishes, while *Aspergillus glaucus* and so on rise in abundance, eventually becoming the primary invasive organisms. Continuing storage for an additional several months leads to a dominance shift towards *Aspergillus flavus* and *Aspergillus albicans* [2,9].

### 2.2. Environmental Requirements for Mold Growth

Mold growth depends on various factors; however, temperature and water activity are vital for their survival and competitiveness. During the early stages of mold development, specific hydrolases (such as β-D-galactosidase, α-D-galactosidase, N-acetyl-β-D-glucosaminidase, β-D-fucosidase, and β-D-xylosidase) significantly influence population establishment and are notably affected by water activity [10]. For most molds, a water activity (a_w_) level of 0.85 or above is conducive to growth [11]; however, when water activity falls below 0.65, it significantly inhibits their growth [12]. Temperature can influence the growth and reproduction of fungi by affecting the activity and function of various enzymes within them. The optimal growth temperature for most fungi is between 20 °C and 30 °C [13], although the range in which they can grow is much broader. Different species of molds have different optimal growth temperatures: the best growth temperature for *Penicillium* and related genera is between 20 °C and 30 °C, and their reproductive structures can produce heat-resistant chain conidia; *Aspergillus* and related molds generally have a higher tolerance to heat, with an optimal growth temperature between 25 °C and 35 °C, making them more dominant in warmer climates [14]; and *Fusarium* and related molds can tolerate a wide range of temperatures, with an optimal growth temperature typically between 25 °C and 30 °C [15]. Additionally, the carbon utilization and niche overlap index (NOI) of mold also respond to changes in water activity and temperature. Research indicates that as water activity increases, the growth rate of mold typically rises. Optimal water activity levels maintain cell structure integrity and enhance growth. Conversely, the impact of temperature on growth rate is relatively minimal and diminishes as water activity levels decrease [16,17,18].

### 2.3. The Detrimental Effects of Mold Infection and the Imperative for Prevention and Management

If molds are not prevented and controlled expeditiously during grain storage, the degree of mold increases significantly. This increase not only causes a gradual rise in the temperature and moisture of the grain, leading to issues such as heat and caking, but the hydrolytic enzymes secreted by molds also decompose fat, proteins, carbohydrates, and other organic substances in the grain. Consequently, a rise in the fatty acid value causes an increase in acidity that accelerates the deterioration of grain quality. Simultaneously, the loss of starch and sugars in the grains reduces weight and increases wastage [4,19,20,21]. In addition, molds can produce secondary toxic metabolites, often referred to as mycotoxins, after infecting grain, mainly including aflatoxins (AFs), fumonisins (FMs), trichothecenes (TRC), ochratoxins (OTs), patulin (PAT), and zearalenone (ZEN), along with their metabolites. Once these toxins contaminate food, they can enter humans and domestic animals through food intake, causing various health hazards. This study has confirmed that these toxins can lead to acute or chronic poisoning in both humans and animals [22].

In summary, preventing and controlling mold in grain storage is extremely important for food security and food safety in China. Effective mold prevention and control measures can lower toxin levels in food and immunize public health. Proper prevention and control can simultaneously reduce food waste and economic losses, thereby promoting the sustainable development of the food industry. Consequently, enhancing mold prevention and control and improving grain storage practices have become top priorities for global food security and safety.

### 2.4. Techniques for Preventing and Controlling Mold

To address mold issues during grain storage, various avenues such as physical prevention and control techniques, chemical interventions, innovative mold inhibitors, and micro–nano technology can be explored. Physical prevention primarily alters non-biological factors within the grain mass to inhibit the physiological processes of mold. Conversely, chemical interventions employ substances to suppress or eliminate mold more rapidly. The latest mold inhibitors utilize sophisticated mechanisms for greater efficacy while aligning with environmental safety and sustainability standards. Micro–nano technology facilitates the controlled release of active ingredients under targeted conditions, demonstrating significant mold suppression while ensuring minimal risk to humans and the ecosystem. Implementing these strategies effectively enhances mold management in grain storage.

## 3. Physical Prevention and Control Technologies

### 3.1. Mechanical Ventilation Technology

**Figure 1:**
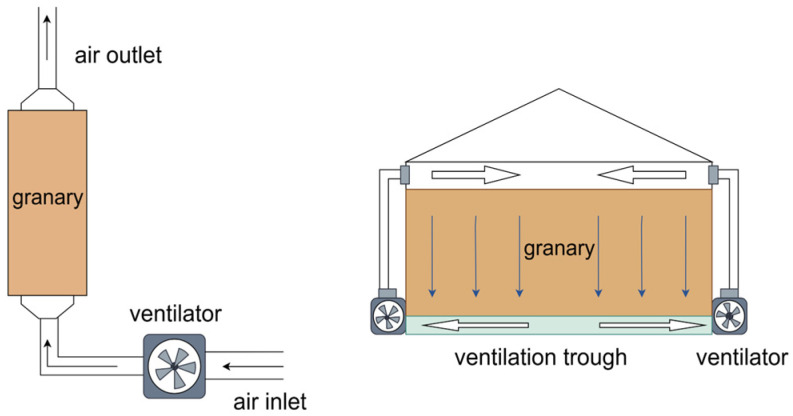
A schematic representation of mechanical ventilation systems.

Mechanical ventilation technology employs fans to exchange hot and cold air within grain piles for cooler, dry air from the outside. This process transforms the internal ecological environment in the pile. By utilizing mechanical ventilation, one can reduce both moisture content and grain temperature, inhibit physiological and metabolic activities of molds, delay quality deterioration, and enhance the safety and stability of grain storage. This technology serves as a vital method for environmental control in grain preservation practices and significantly contributes to maintaining the freshness and quality of stored grains [23] (Figure 1).

The mechanism of mold inhibition through mechanical ventilation is described as follows: after a period of ventilation, the relative humidity of the air within the grain mass falls below critical relative humidity. This discrepancy between the two values broadens over time. Consequently, mold growth and propagation within the grain pile become suppressed. Ongoing ventilation disrupts the temperature conditions necessary for mold development, thereby enhancing the prevention and control of mold [24].

In the process of mold prevention and control through mechanical ventilation, relative humidity, ventilation volume, and different ventilation modes influence their effectiveness. Among these factors, relative humidity impacts grain cooling efficiency, and low humidity airflow can speed up the cooling rate of the grain pile and effectively inhibit mold activity [25]. Ventilation volume is the most critical parameter. In grain ventilation processes, an insufficient ventilation volume may lead to poor cooling effects and a low rate of moisture reduction, making it difficult to effectively control temperature and inhibit mold growth. Conversely, indiscriminately increasing ventilation volume may result in excessively low humidity, causing weight loss in the grain and affecting its quality. This research indicates a critical limit for the ventilation volume needed to manage mold growth. Beyond this boundary, the effect factor ‘F’ reaches a maximum of 1, with no additional improvement in mold suppression noticed. In addition, different ventilation modes vary in mold prevention efficiency. Intermittent ventilation demonstrates greater mold inhibition than continuous ventilation, likely aiding moisture transfer from the inner to the outer layer of wheat. Both pressure-in and suction-out ventilation methods effectively control mold activity. However, suction-out ventilation proves more effective, causing the creation of local negative pressure, accelerating moisture evaporation from wheat. This process deprives molds of necessary moisture, consequently inhibiting their proliferation [26].

### 3.2. Low-Temperature Plasma (LTP) Technology

**Figure 2:**
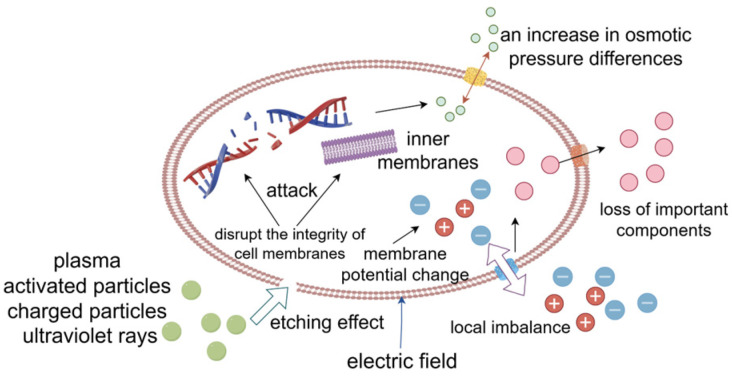
A schematic representation of the mechanism involved in low-temperature plasma sterilization.

Plasma is the fourth state of matter, distinct from solids, liquids, and gases. It is an ionized gas containing a series of active particles, such as electrons, free radicals, and ions. Plasma is categorized by high-temperature plasma and low-temperature plasma [27]. The sterilization mechanism of low-temperature plasma technology (Figure 2) indicates that a plasma generator consists of two key components: high-voltage and low-pressure electrodes. After energization, the two electrodes generate numerous plasma and activated particles, such as oxygen and nitrogen. These activated particles have a certain etching effect and directly attack the structure of microorganisms by disrupting the integrity of their cell membranes, particularly their DNA and inner membranes. Such destruction is due to increased osmotic pressure differences between the intracellular and extracellular environments, ultimately leading to cell death [28]. During the generation of a large amount of plasma between the two electrodes, charged particles and ultraviolet rays are released simultaneously, resulting in damage to cells to some extent, thus enhancing the sterilization effect of low-temperature plasma [29].

This research indicates that the mechanism of low-temperature plasma sterilization also correlates with the generated electric field. This electric field alters the membrane potential of the cell membrane, causing a local disequilibrium in ion flux. Consequently, this disequilibrium leads to the loss of vital cellular components and, in extreme cases, cell death. Notably, the electric field can eliminate cells while preserving the integrity of the cell membrane [30].

The experimental results suggested that the fungicidal effect of this technique was related to treatment time and surface characteristics of the grain. With the extension of time, the number of fungi in the samples treated with the LTP technique decreased significantly, especially within the first fifteen minutes. In addition, the smoother surface of the grains facilitated better contact between the activated particles and the fungi, and the inhibition effect was good. Conversely, grains with rougher surfaces tended to attract more fungi, hindering contact with activated particles and diminishing the inhibitory effect [31].

To sum up, LTP technology is an emerging technology that is more time-saving and efficient, and that can effectively control fungi on the grain surface. Taking corn kernels as an example, the application of LTP technology resulted in a significant decrease in the presence of *A. flavus*. This treatment demonstrated a remarkable inhibitory effect while preserving nutritional integrity [32].

### 3.3. Electron Beam Irradiation Technology

E-beam irradiation is a new technology that uses a stream of electron-beam rays generated by an electron accelerator to penetrate a substance and initiate physical, chemical, and biological changes for sterilization [33]. In this process, water molecules in the substance receive energy to produce free radicals H·and·OH, which are involved in chemical changes in biological macro-molecules, leading to chain breakage and reorganization, thus causing physical and chemical reactions within the substance and deteriorating enzyme activity [34,35]. Furthermore, the irradiation energy also disrupts genetic fragments and proteins of microorganisms, thereby inhibiting their growth and reproduction [36,37].

Studies have shown that electron beam irradiation can significantly inhibit the growth of molds and effectively degrade mycotoxins, which has good application prospects [38,39]. Taking *A. flavus* as an example, electron beam irradiation causes noticeable wrinkling and twisting of its mycelium. Most spores exhibit similar wrinkling. Moreover, the chitin content associated with the cell membrane and wall decreases, while chitinase activity diminishes and malondialdehyde levels rise, resulting in cellular structural damage. Furthermore, electron beam irradiation disrupts the intracellular tricarboxylic acid cycle in *A. flavus*. This disruption leads to increased pyruvate dehydrogenase enzyme activity, yet insufficient initiation of the tricarboxylic acid cycle, causing mitochondrial failure. Consequently, this failure reduces electron transfer efficiency and elevates intracellular reactive oxygen species levels, leading to membrane disruption, enzyme inactivation, and apoptosis. It is also found that the inhibitory effect is more pronounced with increased irradiation dose [40].

## 4. Chemical Prevention and Control Technologies

Chemical prevention and control technologies employ chemical agents to inhibit mold growth and reproduction, thus effectively controlling the further development of mold activities in grain storage within a short period. Based on the specific application of chemical reagents, this technology can be divided into three types: solid mold inhibitor prevention and control, liquid mold inhibitor prevention and control, and chemical gas conditioning prevention and control.

### 4.1. Solid Inhibitors for the Prevention and Control of Molds

Solid mold inhibitors usually include certain organic acids and their salts, such as benzoic acid and benzoate, sorbic acid and sorbate, and sodium diacetate. Among them, benzoic acid exerts its anti-mold effect by inhibiting the activity of microbial-specific enzymes. Additionally, it has been found that benzoic acid and benzoate show good inhibitory effects against yeasts, molds, and some bacteria, making them widely used [41]. Sorbic acid and its salts can destroy the enzyme system by combining with the sulfhydryl group in the enzyme system and then have an effective inhibitory effect [42]. Sodium diacetate is a molecular composite compound of acetic acid and sodium acetate, which has a strong inhibitory effect on molds [43]. The acetic acid molecule within the compound can lower the pH value of substances. Due to its excellent compatibility with ester compounds, sodium diacetate can easily penetrate cell walls and enter cells. This process disrupts intercellular enzyme interactions, promotes protein denaturation, and alters cell morphology and structure. As a result, this leads to mold dehydration and ultimately inhibits mold growth [44].

In addition, studies have shown that solid mold inhibitors can significantly enhance their inhibitory effect when applied in combination with other mold inhibitors. Specifically, at a pH of 4.5, sodium benzoate and cinnamon essential oil effectively inhibit the growth of *A. flavus* synergistically. Secondly, the pH of a solution also plays an important role in inhibiting mold growth. By optimizing the pH level, one can amplify the synergistic effect, thereby more effectively suppressing mold growth [45]. This discovery offers a significant theoretical foundation for the future development of innovative composite mold inhibitors.

### 4.2. Liquid Inhibitors for the Prevention and Control of Molds

Liquid antifungal agents are widely used in grain storage mold prevention because of their good performance, with active ingredients that include propionic acid, hexanal, n-hexanol, and so on. Propionic acid serves as a colorless, volatile liquid mold inhibitor. Its inhibition mechanism operates as follows: the active molecules of propionic acid exist in a non-dissociative form, creating high osmotic pressure outside the mold cells [46]. This pressure induces dehydration within the cells. However, mold requires a certain level of cellular moisture to sustain metabolic activities. When dehydration occurs, its physiological functions will be impaired, leading to the loss of reproductive capacity [47]. Furthermore, propionic acid active molecules not only function effectively in the extracellular environment but also penetrate the cell wall of molds. This action inhibits enzyme activity within the cells, thereby preventing mold reproduction [48]. Both hexanal and n-hexanol are effectively used in fumigation treatments to inhibit mycelial growth and delay spore formation. The key aspect of this process is that when these substances are emitted during fumigation, they can infiltrate fungal cells and disrupt the normal operation of organelles, thus hindering spore development. Among them, hexanal shows better advantages with respect to inhibiting molds in stored grains; when its concentration reaches 1.66 mmol/L, it can completely inhibit the activity of molds in high-moisture grains with relatively little effect on grain quality [26].

**Figure 3:**
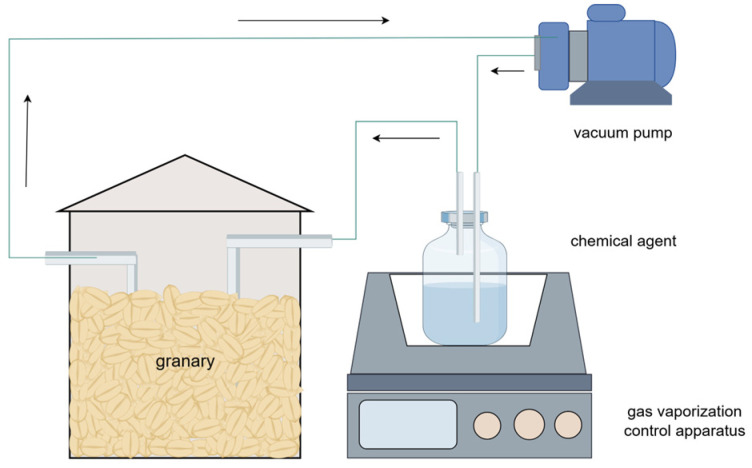
A schematic representation of the circular flow fumigation process.

Research indicates that the bacteriostatic efficacy of hexanal varies with different fumigation modes. In natural volatilization fumigation, the slow diffusion of the agent obstructs uniform distribution, limiting its bacteriostatic potential. Conversely, cyclic flow fumigation (Figure 3) effectively enhances the rapid and uniform distribution of the agent within the grain pile. This allows grain particles to contact the agent earlier, thus extending the action’s duration and improving its fumigation effect [49]. Furthermore, hexanal has a rapid onset of action against molds and can inhibit or kill growing molds in a short period of time, which makes it very suitable for the fumigation of open piles. Unlike traditional fumigation agents, hexanal does not require sealing within the grain silo during application, thus simplifying operational procedures [26]. Additionally, utilizing this fumigant minimizes potential safety risks and mitigates adverse environmental effects.

In addition, a study revealed that both the addition of liquid mold inhibitor stock solution and diluted solution could effectively inhibit the growth of *A. niger*, *Rhizopus*, and *Penicillium*. After further analysis, it was observed that the diameter of the inhibition zone for the stock solution exceeded the diluted solution, indicating a significant positive correlation between the inhibition effect of liquid mold inhibitor on molds and its concentration [50]. This provides an important basis for optimizing the use of mold inhibitors and helps achieve the best prevention and control effects by selecting the appropriate concentration.

### 4.3. Chemical Gas Conditioning

Gas mold inhibitors, including ozone and chlorine dioxide, prevent the growth of molds by releasing active ingredients to protect grains. Ozone is prevalent in microbial inhibition and mold prevention in grains. Its inhibitory mechanisms include (1) oxidizing key cellular components, which disrupts microbial integrity; (2) altering cell permeability by interacting with lipoproteins and lipopolysaccharides, causing internal leakage and cell lysis; and (3) directly targeting organelles and nucleic acids, disrupting vital life processes and resulting in microbial death [51]. In addition, ozone is also effective in degrading mycotoxins such as aflatoxins, ochratoxins, and fumonisins [52]. Nevertheless, chlorine dioxide inhibits mold by adsorbing onto and penetrating microbial cell walls, entering the cell, and inducing redox reactions in amino acids, which leads to their decomposition and ultimately disrupts microbial protein synthesis, causing microbial death [53].

Studies have shown that the fungicidal effect of ozone is closely related to treatment concentration and time. Taking stored corn as an example, after ozone treatment, the inhibition rate of fungi showed a trend of rising and then leveling off with the increase in ozone treatment concentration. Within a specific range, increased ozone concentration enhances the inhibitory effect on corn fungi. Furthermore, the inhibition rate showed an increasing and then decreasing trend with the prolongation of the treatment time. When the ozone treatment time is less than 40 min, the inhibition rate shows an increasing trend, peaking at 57.62% at the 40 min mark. Beyond this duration, the inhibition rate gradually declined. This phenomenon may be related to the fact that the half-life of ozone is 15 to 30 min. With the extension of the treatment time, the stability of ozone decreases, causing it to decompose into oxygen [54]. This process leads to an increase in the number of microorganisms, thus reducing the antibacterial rate [55]. In addition, ozone treatment leads to a reduction in the moisture content of corn, an increase in brightness, an elevation in electrical conductivity, a decrease in starch content, an increase in malondialdehyde content, and a rise in fatty acid value, but it has almost no effect on the germination rate [56]. Chlorine dioxide, on the other hand, has a good inhibitory effect on *A. flavus*. Its action time and concentration are the main factors affecting the germination of *A. flavus* spores when the high moisture content in corn is used as a carrier. Specifically, prolonging the bactericidal duration at low concentrations or administering short treatments at high concentrations can effectively inhibit the germination of *A. flavus* spores [57].

## 5. Innovative Mold Inhibitors for Grain Storage

A new kind of mold inhibitor is a chemical preparation developed to effectively deal with the problems caused by mold during grain storage. It employs an innovative mold inhibition mechanism, demonstrating enhanced efficacy in curbing mold growth and reproduction. At the same time, it emphasizes ecological safety and corresponds with sustainable development objectives, reducing negative impacts on the environment and public health. This type of mold inhibitor leverages the beneficial effects of microbial antagonistic bacteria in nature, along with the metabolites of microorganisms and the rich antimicrobial components in natural plant extracts. Additionally, it effectively prevents the occurrence of mold and can be divided into microbial-derived mold inhibitors and plant-derived mold inhibitors according to their origins.

### 5.1. Microbial-Derived Mold Inhibitors

The active components of microbiologically derived mold inhibitors consist of volatiles generated by specific fungi, such as *Bacillus* and *Streptomyces alboflavus* TD-1, along with recombinant proteins, like Puroindoline A. Additionally, mold inhibitors can take advantage of the positive effects of microbiologically antagonistic bacteria in nature to carry out preventive measures and control measures. For example, by introducing non-toxic *A. flavus*, the growth of toxic *A. flavus* and the production of its toxin can be effectively inhibited, thus achieving a prevention and control effect.

Volatile substances produced by specific fungi, such as *Bacillus* and *S. alboflavus* TD-1, not only have a highly effective anti-mold effect but also demonstrate safety in application. Among them, the volatile substances produced by *Bacillus* can effectively inhibit mycelial growth and spore germination of mold, alter its colony morphology, reduce pigment production, and affect the micro-structure to achieve the effect of mold inhibition [58]. Research indicates that, among *Bacillus* volatile compounds, isooctanol and isobutyric acid are crucial for reducing paddy mildew. Their mold inhibition rate significantly surpasses that of other compounds, and they have broad-spectrum mold inhibition for seven common molds (*A. flavus*, *A. niger*, *Aspergillus ochraceus*, *F. graminearum*, *Rhizopus* spp., *Rhizomucor pusillus*, and *Penicillium oxalicum*). Moreover, research has found that when isobutyric acid and isooctanol are mixed in a 1:2 ratio, the mixture effectively controls the increase in fatty acid values in paddy while efficiently preventing molds. A one-way ANOVA review found that the efficacy of mixture control on mold quantity and fatty acid values became more pronounced as the moisture content of the paddy increased. The most significant control effect occurs at a fumigation temperature of 30 °C, and prolonged fumigation time further enhances isooctanol effectiveness in managing mold quantity and fatty acid values in paddy [59]. Additionally, among the volatiles produced by *S. alboflavus* TD-1, 1-octen-3-ol demonstrates a notable inhibitory effect on *A. flavus* and can reduce the production of *A. flavus* AFB1, which prevents and controls *A. flavus* through the following mechanisms: Firstly, 1-octen-3-ol inhibits *A. flavus* spores from germinating and reduces the chances of infection; secondly, it can further inhibit *A. flavus* by destroying the integrity of the membrane and mitochondrial membrane, leading to cell failure [60].

The recombinant protein Puroindoline A is obtained by constructing a prokaryotic expression vector for the Pina gene followed by purification after successful expression in *E. coli* [61]. This research indicates that the recombinant protein PINA has a certain inhibitory effect on common grain storage molds. Notably, *A. glaucus* serves as a typical dry grain storage mold, prosperous under low moisture conditions. Therefore, it is considered a “warning species” for early mold development in grain storage, making effective inhibition of its growth crucial for mold prevention and control. After treatment with recombinant protein PINA, *A. glaucus* mycelium exhibits abnormal morphology changes, including flattening and creasing. Simultaneously, the presence of protein is detected in the mycelium. Laser confocal observation reviewed a compromise in cell membrane integrity, a reduction in mitochondrial membrane potential, and evidence of DNA damage in the treated mycelial cells. These physiological changes indicate that the recombinant protein PINA hinders the growth of *A. glaucus* and plays an effective inhibitory role. Additionally, its inhibitory effect is affected by a variety of physicochemical factors. Firstly, the concentration of recombinant protein PINA shows a positive correlation with its inhibitory effect. In other words, as the concentration increases, the inhibitory effect becomes more pronounced. Secondly, regarding temperature, it exhibits enhanced bacteriostatic activity at low to moderate temperatures. However, its inhibitory effect diminishes when temperatures exceed 50 °C. Furthermore, recombinant protein PINA exhibits effective bacteriostatic activity in mildly acidic and mildly alkaline conditions, but its bacteriostatic activity decreases significantly under strong acid or strong alkali conditions [62]. Thus, one can effectively regulate the concentration, ambient temperature, and pH of recombinant protein PINA to enhance its bacteriostatic properties and broaden its potential applications in grain storage.

Microbial-derived mold inhibitors can also be applied for mold prevention through competitive inhibition by non-toxin-producing molds. *A. flavus* is a prevalent mold found in grain storage, and its infestation not only leads to quality degradation but also heightens the risk of aflatoxin contamination, thereby threatening food security and public health [63]. Studies have shown that non-toxic strains of *A. flavus* can significantly suppress the proliferation of toxin-producing *A. flavus* and its toxin synthesis [64]. Furthermore, even the minimal presence of non-toxic *A. flavus* can produce a better biological control effect. This type of control primarily involves the suppression of *A. flavus* spore production and the radial expansion of colonies, thereby inhibiting the growth of *A. flavus* and consequently reducing toxin production. During this process, the expression of genes related to toxin biosynthesis (aflE, aflL, and aflP) and spore formation (abaA, con6, and vosA) diminishes. In addition, it was observed that both the inoculation time and ratio significantly influence the inhibition effect. Specifically, when the delay in inoculation does not exceed two days, the introduction of non-toxigenic *A. flavus* effectively inhibits toxin-producing *A. flavus*, irrespective of the priority effect. Moreover, the most effective inhibition of toxin-producing *A. flavus* occurs in co-culture experiments involving a single non-toxigenic *A. flavus* strain with toxin-producing *A. flavus*, particularly when the inoculation of non-toxigenic *A. flavus* at a spore concentration ratio of 1:50 is delayed by one day. In co-culture experiments with mixed non-toxigenic and mixed toxigenic *A. flavus*, optimal inhibitory effects are achieved when inoculation performs simultaneously at a spore concentration ratio of 1:2 [65]. Therefore, through reasonable inoculation strategies and ratio adjustments, non-toxigenic *A. flavus* shows good application prospects in mold prevention and toxin production inhibition.

### 5.2. Plant-Derived Mold Inhibitors

Plant-derived mold inhibitors effectively prevent mold growth by utilizing the rich array of antimicrobial compounds existing in natural plant extracts. These active constituents encompass terpenoids, such as gaseous cinnamaldehyde, citral, and linalool, as well as volatile fatty acid derivatives, including octanol, nonanol, hexanal, and heptanal. Notably, essential oils derived from plants are the most frequently employed formulation due to their high efficacy, non-toxicity, and remarkable antibacterial properties.

**Figure 4:**
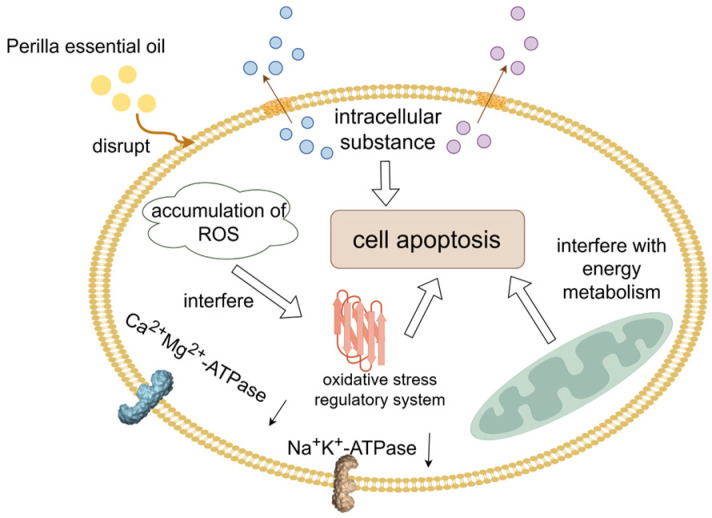
A schematic representation of the bacteriostatic mechanism of Perilla essential oil.

The essential oil of Perilla exhibits broad-spectrum antimicrobial properties and demonstrates significant potential, particularly in the prevention and control of grain contamination caused by *A. flavus* [66]. Perilla is an annual erect herb belonging to the genus Perilla in the family Labiatae with its essential oil primarily extracted from the leaves [67]. Research has indicated that Perilla essential oil exhibits a good inhibitory effect on *A. flavus*, effectively inhibiting spore germination and mycelial growth. The inhibition occurs through a multi-targeted, multi-pathway mechanism, which includes the disruption of cell membrane integrity, leading to the effusion of cellular contents and increased membrane permeability. This disruption reduces the activity of Ca^2+^Mg^2+^-ATPase and Na^+^K^+^-ATPase. Additionally, it induces the accumulation of reactive oxygen species (ROS) within the organism, exacerbating oxidative stress damage by impairing the antioxidant enzyme system and interfering with the organism’s oxidative stress regulatory system. Furthermore, it inhibits the glycolytic pathway to ensure energy supply for the mold post-injury. This process activates amino acid metabolism, pyruvate metabolism, fatty acid degradation, glyoxylic acid metabolism, and the tricarboxylic acid cycle, all of which help to maintain normal energy metabolism. It also up-regulates proteins associated with transcription and translation, promoting the synthesis of enzyme proteins in the body or compensating for protein effusing following cell membrane damage (Figure 4). Moreover, it is determined that the minimum inhibitory concentration (MIC) of Perilla essential oil against *A. flavus* is 0.4 μL/mL, with the spore germination rate and dry weight of *A. flavus* showing a negative correlation with the volume concentration of essential oil as the concentration increases. Throughout the storage period, fumigation treatment with Perilla essential oil significantly inhibits the increase in the fatty acid value of rice, effectively reduces the malondialdehyde content in rice, and maintains the structure and function of the rice organism’s membrane. This effectively delays the quality deterioration trend of high-moisture rice [68]. These findings provide strong support for the application of Perilla essential oil as a natural mold inhibitor in grain security storage.

Terpenoids, including gaseous cinnamaldehyde, citral, and linalool, are principal constituents of plant essential oils and exhibit notable inhibitory effects on molds affecting stored grains. Among them, gaseous cinnamaldehyde has a strong inhibitory effect on *Aspergillus niger* HY2. Its mechanism of action primarily involves the disruption of the cell wall, allowing it to penetrate intracellularly, thereby inhibiting ergosterol synthesis, damaging the cytoplasmic membrane, and increasing cell membrane permeability. This results in the leakage of many substances, ultimately disrupting the homeostatic environment of microbial cells. Concurrently, it leads to a decrease in mitochondrial membrane potential and the activity of ATP and SDH enzymes, which inhibits ATP synthesis, and ultimately causes metabolic disorders in *A. niger* HY2, leading to the organism’s death. These studies have demonstrated that the application of gaseous cinnamaldehyde in paddy storage treatment offers significant benefits: Firstly, it effectively inhibits mold growth, thereby reducing the risk of contamination and ensuring safe storage; and secondly, it maintains the good quality of paddy, lowers fatty acid levels, and minimizes fat decomposition [69]. Gaseous cinnamaldehyde also demonstrates a strong inhibitory effect in controlling aflatoxin contamination. Short-term treatment with gaseous cinnamaldehyde disrupts both the cell surface and internal structure, leading to compromised cell membrane integrity, inducing ROS accumulation and lipid peroxidation, and promoting increased antioxidant enzyme activities. Crucially, after the removal of gaseous cinnamaldehyde, although cell membrane function can be restored, ROS and the lipid peroxidation product MDA are scavenged, and antioxidant enzyme activities return to normal levels. However, the presence of apoptosis-related cavitation in *A. flavus* cells prevents them from returning to normal, while the disordered mitochondrial membrane potential and inhibited ATPase activity remain irreversibly damaged [70]. Furthermore, it was observed that the impact of short-term gaseous cinnamaldehyde treatment on the growth and toxin synthesis of *A. flavus* is concentration-dependent, indicating that damage caused by low concentrations can be recovered after the removal of gaseous cinnamaldehyde, whereas damage from high concentrations is irreversible [71].

Citral is derived from a variety of sources and is predominantly found in lemon grass oil (approximately 70–80%), litsea cubeba oil (80%), lemon oil, and citrus leaf oil [72,73]. It exhibits broad-spectrum antibacterial activity and demonstrates a significant bactericidal effect on molds [74]. Research has indicated that citral can markedly inhibit the colony growth and spore germination of *F. graminearum*—a common pathogen in wheat storage—resulting in irreversible damage to its conidia and mycelium morphology, while also significantly reducing the accumulation of the DON toxin. Treatment with citral also decreases the ergosterol content in the cell membrane of *F. graminearum*, simultaneously triggering membrane lipid peroxidation, which increases the extracellular malondialdehyde content, thereby damaging the cell membrane. This damage leads to the failure of intracellular substances, ultimately resulting in cell death. This process represents one of the key mechanisms through which citral exerts its antibacterial activity. Furthermore, citral disrupts both the antioxidant and the energy metabolism system, causing severe cellular damage and inhibiting mold growth. Specifically, citral primarily achieves its inhibitory effects by suppressing pyruvate metabolism, tyrosine metabolism, and the metabolism of glycine, serine, and threonine, among other pathways. The research also found that the method of applying citral through a forced circulation system outside the grain pile for mold prevention in wheat allows for flexible adjustment of the dosage based on circulation time or frequency. This method does not introduce any residual mold inhibitors into the stored wheat, making it a relatively ideal chemical treatment for mold prevention in stored wheat [75].

Linalool is the primary bacteriostatic component of peppercorn pericarp volatiles, which can effectively inhibit the growth of *A. flavus* during grain storage. Studies have demonstrated that linalool induces damage to the cell membrane of *A. flavus*, leading to the diminishing of cell content, thereby affecting its survival. Further metabolomics reviews, along with physiological and biochemical experiments, have observed that linalool inhibits the growth of *A. flavus* through various mechanisms, including the disruption of cell membrane permeability and integrity, interference with the TCA cycle, ABC transport, and the induction of mitochondrial dysfunction and oxidative stress [76]. The transcriptomic and physiological results indicate that linalool primarily inhibits spore germination by disrupting the cell membrane, causing mitochondrial dysfunction and DNA damage, ultimately leading to autophagy [77].

The active ingredients in plant-derived mold inhibitors also include octanol, nonanol, hexanal, and heptanal. Studies have demonstrated that both octanol and nonanol can completely inhibit the proliferation of *A. flavus* mycelial at a concentration of 0.2 μL/mL. Specifically, octanol disrupts the energy supply by compromising the cell membrane integrity of *A. flavus*, leading to mitochondrial dysfunction. It also interferes with the MAPK signaling pathway, disrupting processes involved in transferring genetic information, such as DNA replication and transcription, and inducing autophagy pathways, ultimately resulting in cell death. Nonanol has the potential to compromise the integrity of the cell wall and membrane of *A. flavus*. It inhibits the TCA cycle, disrupts fatty acid and amino acid metabolism, and interferes with oxidative phosphorylation. Additionally, it affects the processes involved in transmitting genetic information, including DNA replication and transcription. Furthermore, nonanol activates both the antioxidant pathway and autophagy, ultimately resulting in the apoptosis of *A. flavus* cells [78]. Additionally, hexanal and heptanal have demonstrated the ability to inhibit the growth of *A. flavus* and affect its normal physiological and biochemical properties, thereby exhibiting a beneficial effect on preventing and controlling *A. flavus* in cereals. Among these, the inhibitory effect of hexanal primarily arises from its interference with the main energy metabolic pathways within *A. flavus*, which is reflected in alteration of the cell membrane composition, deficiency in mitochondrial function and energy metabolism, disruption of oxidative phosphorylation, interference with genetic information transmission, elevation of autophagy levels, and induction of dysfunctions in the antioxidant system. Additionally, physiological and chemical experiments have confirmed its effectiveness in inducing apoptosis in *A. flavus* spores. Conversely, heptanal operates by regulating metabolic pathways, specifically by disrupting the cell wall and membrane of *A. flavus*, blocking DNA replication, inhibiting the cell cycle, and mediating the activation of AMPK to suppress the growth of *A. flavus* [79].

## 6. Micro–Nano Prevention and Control Technology

**Figure 5:**
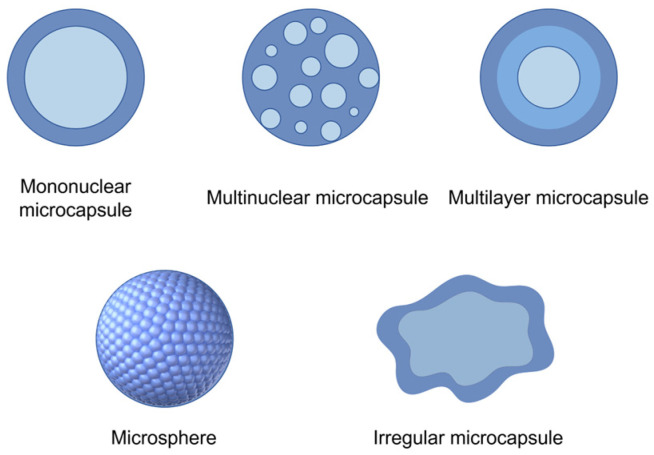
The main structures of microcapsules.

Microencapsulation technology employs polymers as carriers to encapsulate essential oils or their active ingredients that require protection into tiny particles, with diameters measured in microns or nanometers. This process effectively immobilizes and safeguards the core ingredient, besides facilitating controlled release under specific conditions, prolonging retention time, and enhancing antimicrobial properties [80]. Due to the selection of wall materials, core materials, and preparation methods, the structure of microcapsules is different and is mainly divided into five types: mononuclear microcapsules, multinuclear microcapsules, multilayer microcapsules, microspheres, and irregular microcapsules [81] (Figure 5). Among them, mononuclear microcapsules are the most fundamental type, characterized by a single core enveloped in a layer of wall material. In contrast, multinuclear microcapsules possess multiple internal cores and primarily generate through the encapsulation of emulsified substances. Multilayer microcapsules build upon the structure of mononuclear microcapsules by incorporating one or more additional layers of wall material, thereby enhancing their protective properties and controlled-release capabilities [82,83]. Furthermore, the cores of microspheres and irregular microcapsules typically encapsulate as particles or droplets within the wall material, lacking a distinct membrane structure [84].

Carvacrol–chitosan nanoparticles employ a technology that effectively inhibits *F. graminearum*. Research indicates that the treated *F. graminearum* mycelium exhibits deformation and affective disorder, with compromised plasma membrane integrity leading to the failure of cellular content. This disruption adversely affects normal bacterial growth, culminating in cellular damage and death, inhibiting *F. graminearum* proliferation. Notably, the minimum inhibitory concentration (MIC) of carvacrol–chitosan nanoparticles is determined to be 125 μg/L. When the concentration exceeds 2 × MIC, complete inhibition of *F. graminearum* growth is observed, demonstrating a clear concentration-dependent inhibitory effect. Consequently, carvacrol–chitosan nanoparticles facilitate the sustained release of carvacrol, extending the release duration and enhancing the stability of its antibacterial activity [85].

In conclusion, micro–nano technology enables the effective embedding and controlled release of active ingredients. It not only achieves superior prevention and control outcomes, but it also offers innovative insights and directions for advancing mold prevention in grain storage.

## 7. Economic Costs of Prevention and Control Technologies

Mechanical ventilation equipment, solid mold inhibitors, and liquid mold inhibitors are relatively inexpensive in terms of price, while the cost of chemical gas control is higher. The overall price of micro–nano technology depends on the selection of wall and core materials as well as the complexity of the preparation process, making it often quite expensive (Table 1). Additionally, some other technologies are still in the experimental stage and require further research and optimization.

## 8. Prospect

Despite the progress in prevention and control technologies and the development of novel grain storage mold inhibitors, several issues still warrant further investigation. Primarily, external factors, such as fluctuations in temperature and humidity during grain storage, can significantly influence the efficacy of mold prevention. Therefore, it is crucial to explore the stability and antimicrobial activity of mold inhibitors under varying temperature and humidity conditions and the strategies for regulating the grain storage environment to optimize the performance of these inhibitors.

Additionally, existing research on the active components of mold inhibitors predominantly involves small-scale storage simulations and focuses on single experimental strains. However, the storage environment is extensive, with complex and variable environmental factors in practical applications. It is imperative to broaden the scope of research and conduct comprehensive studies on inhibitory activities and mechanisms against a diverse range of grain storage molds. Thus, the limitations of each prevention and control technology in specific scenarios require further in-depth exploration. Moreover, the impact of each prevention and control technology on grain quality while inhibiting mold necessitates further examination. The potential use of composite mold inhibitors should also be investigated in terms of their synergistic effects on mold inhibition, aiming to enhance the overall efficacy of mold suppression.

## Figures and Tables

**Table 1:** Economic costs of some prevention and control technologies.

Technology	Consumable Costs (Euro/kg)	Instrument Costs (Euro/Unit)
Microencapsulation	chitosan: 1.31–6.54; carvacrol: 39.22–78.43	-
Mechanical ventilation	-	ventilator: 261.44–1307.19
Solid mold inhibitors	benzoic acid: 1.05–1.91; sorbic acid: 2.61–3.92; sodium diacetate: 1.31–1.96;	-
Liquid mold inhibitors	propionic acid: 1.31–1.96; hexanal: 3.92–6.54; n-hexanol: 2.61–4.58	-
Chemical gas conditioning	-	medium-sized ozone generator: 6535.95–26,143.79; medium-sized chlorine dioxide generator: 2614.38–13,071.9

## Data Availability

No new data were created or analyzed in this study. Data sharing is not applicable to this article.
